# Personal and Organized Charity

**Published:** 1875-04

**Authors:** 


Personal and Organized Charity.—The problem of harmonizing official and voluntary charity is an important one. We do not find fault with public institutions of charity. In many such there is honest fidelity and well-meant endeavor. But we must bear in mind that they are subject to political influence. The places of trust in an almshouse are often merely political rewards. Changes in administration bring changes in those who care for the poor, and consequently people of little experience are placed over them, who are easily prejudiced and misled. There is a vast work to be done—that of rescuing and redeeming children from manifold evils of institutional training. The problem of pauperism is not simply of to-day, but we must prevent the pauper of the present from producing two hundred or six hundred in the future. We must not only arrest their progress now, but their multiplication by an arithmetical progression terrible to contemplate. It is well to recognize that there are two classes—the redeemable and the irredeemable, the hopeful and the hopeless. There is a large and noble work for those who love children and their country. To place the 1600 children now on Randall's Island in decent and orderly homes would be a proportion of one child to every fifty families in the State. A casual glance at this work and its dimensions would produce hopelessness and despair. As you approach closer to actual want, it is ennobled and transfigured.

---

In business, believe in travelling step by step; don't expect to get rich in a jump.

The Relief of Want.—The relief of want is founded not alone in humanity, but in the preservation of human society. Wants result from crime, and all the evils that threaten humanity. We want to know, not what to do, but how to do it. No person's position is so assured that he or his descendants may not fall into calamity. In some countries, mendicity and vagrancy do not exist, or they are so limited as not to be perceivable. It is so in Holland and Northern Germany, while in Great Britian, Ireland, Scotland, France, Spain, and our own country, they abound. If the world were filled with vagrants, human society would be impossible. They should be brought to the point where they must select between labor and starvation. This is difficult, because there are periods when even the industrious can not obtain employment. We have no great faith in the power of governments or laws to reach such classes. We must understand the nature of the problem, and also learn from foreign lands, where poverty is rare. The only course that can be pursued with children is to secure for them homes in families.

---

One of the causes of sickness, suffering, and premature death, is the exclusion of sunlight. Sunlight is necessary to the health of mankind; it is necessary to the health of every kind of animal which exists on the face of the earth. It is also necessary to the health of all the plants, flowers, and trees, that live in the fields and forests.

---

He who undertakes too much succeeds but little.

